# Creation of an Automated Fluorescence Guided Tumor Ablation System

**DOI:** 10.1109/JTEHM.2021.3097210

**Published:** 2021-07-21

**Authors:** Matthew Tucker, Guangshen Ma, Weston Ross, Daniel M. Buckland, Patrick J. Codd

**Affiliations:** 1 Department of Mechanical Engineering and Materials ScienceDuke University 3065 Durham NC 27708 USA; 2 Department of NeurosurgeryDuke University 3065 Durham NC 27708 USA; 3 Division of Emergency MedicineDuke University 3065 Durham NC 27708 USA

**Keywords:** Automated surgery, cancer detection, surgical sensor fusion, tumor ablation

## Abstract

Objective: Create a device that improves the identification and extent of resection at the interface between healthy and tumor tissue; ultimately, using this device would improve surgical outcomes for patients and increase survival. Methods: We have created a contactless tumor removal system that utilizes endogenous fluorescence feedback to inform a laser ablation system to execute autonomous removal of phantom tumor tissue. Results: This completely non-contact surgical system is capable of resecting the tumor boundary of a tissue phantom with an average root mean square error (RMSE) of approximately 1.55 mm and an average max error of approximately 2.15 mm. There is no difference in the performance of the system when changing the size of the internal tumor from 7.5-12.5 mm in diameter. Discussion: Future research steps include creating a more intelligent spectral search strategy to increase the density of points around the resection boundary, and to develop a more sophisticated classifier to predict pathologic diagnosis and tissue subtypes located regionally around the tumor boundaries. We envision this device being used to resect the boundaries of tumors identified by exogenously delivered tumor-labeling fluorophores, such as fluorescein or 5-ALA, in addition to approaches relying on autofluorescence of endogenous fluorophores.

**Clinical and Translational Impact Statement:** The automated system has the potential to increase the identification and extent of resection for cancerous tumors. These improvements can increase the survival for brain tumor patients.

## I. Introduction

Neurosurgical removal of a brain tumor is typically performed through a craniotomy, which includes the temporary removal of a portion of the skull in order to access the intracranial environment [1]. Pre-operative imaging modalities such as computed tomography (CT), magnetic resonance imaging (MRI), and even angiography can be combined with external reference systems to aid the surgeon in designing the location of this craniotomy for each patient, and for localizing the underlying tumor for surgical resection [2]–[4]. The standard of care in neurosurgery employs mechanical dissection tools such as scalpels and aspirators. However, these mechanical methods of tumor resection are still ultimately limited by the precision of the human operator, highlighting the need for ultra-precise surgical tools.

Fluorescence spectroscopy is another commonly used intraoperative sensing modality that assists the surgeon in localizing a pathologic tissue. The process of using fluorescence to aid in the localization and removal of tumors is called fluorescence guided surgery (FGS) [5]. FGS is an important augmentation to the intraoperative imaging data stream available to the surgeon and facilitates maximal safe surgical resection, especially at the tumor margins where every millimeter of tissue resected outside the tumor region itself could prove devastating neurologically for the patient. FGS allows localization of tumor margins intraoperatively and in real-time. Additionally, FGS is less impactful to the surgical workflow and less expensive than other intraoperative sensing methods such as intraoperative MRI (iMRI) [6], [7].

One popular FGS technique in neurosurgery requires the administration of 5-aminolevulinic acid (5-ALA) to aid in the identification and treatment of brain tumors [8]. 5-ALA is administered to the patient before surgery and accumulates in malignant brain tumors [9]. The accumulated 5-ALA is metabolized into protoporphyrin IX (PpIX). PpIX is a fluorescent metabolite that can reveal the presence of a brain tumor after excitation with 405 nm light [8], [9]. The use of 5-ALA has resulted in higher rates of complete resection in malignant gliomas when compared to resection using traditional white-light [8].

Although 5-ALA has demonstrated value in the operating room, it can result in side-effects such as false-positive tumor identification and resulting neurologic deficits from over-resection, temporary skin sensitivity, or allergic reaction [10]. Furthermore, the clearance rate of PpIX requires accurate timing and limits the length of utility during surgery. Because of this, there is a clear need for a rapid, easily applied real-time intraoperative tumor identification method that does not rely on exogenously administered compounds. Tissue autofluorescence stands to serve as a viable solution for this need. A substantial body of work has been created around how measuring autofluorescence taps into the metabolic differences between healthy and neoplastic cells, and can be used to aid in the diagnostic differentiation of the tissue [11], [12]. The metabolic phenomenon that is exploited in this imaging technique is called the Warburg effect [13].

The Warburg effect, discovered by Otto Von Warburg in the early 20^th^ century, describes how healthy and neoplastic tissue differ in producing energy for a cell [13]. Neoplastic cells utilize fermentation, in the presence or absence of oxygen, to generate energy [13]. This difference in energy generation strategies leads to differing absorption and scattering properties across tissues and differing concentrations of coenzymes and cofactors crucial between healthy and neoplastic tissue. Some of these molecules, such as nicotinamide adenine dinucleotide (NADH) and flavin adenine dinucleotide (FAD), are fluorophores. Through this principle, it is possible to use fluorescence spectroscopy to detect differences in endogenous fluorescence intensity between neoplastic and healthy cells. There are a number of groups that have shown that the induced fluorescence can be used to identify the differences between neoplastic and healthy tissue [14]–[16]. This opens the possibility for endogenous fluorescence to serve as an sensory input to guide surgical resection of pathologic tissues while avoiding removal of normal healthy tissue, which is demonstrated herein.

Surgical robotics have long been considered as a potential avenue to improve the accuracy and precision of surgical action to accomplish the surgical goal, while improving the safety and clinical outcome for the patient [17]. The first robotic surgical procedure in the modern era was conducted in 1988 [18]. Kwoh *et al.* developed the technology for the procedure with the intention of conducting a neurosurgical biopsy with greater accuracy [18]. Other robots, such as the steady-hand robotic system developed by researchers at Johns Hopkins University, was created to reduce small movements in position due to physiological hand tremors experienced by microsurgeons [19].

Not all robots introduced focus on stabilizing the hand movements of surgeons. The TumorCNC, developed by the Brain Tool Laboratory at Duke University, was developed with the vision to create a system that could leverage computer numerical control (CNC) and photonic solutions to remove tumorous tissue from the brain [20]. The TumorCNC utilizes a $10.6~\mu m~CO_{2}$ laser, a distance triangulation sensor, and two-dimensional scanning galvanometer mirrors to remove tissue via ablation without the need for a surgeon to physically hold a device. Traditionally, many robots have been designed to be supervised and controlled by the surgeon [21]. However, a recent invention, known as the Smart Tissue Autonomous Robot (STAR), outperformed human surgeons at repairing (stitching) the small intestines of a pig [22].

Towards improving the precision in neurosurgical oncology, we have created a non-contact, automated tumor ablation device that relies on fluorescence spectroscopy to autonomously differentiate healthy tissue from neoplastic tissue. This device, which is a unification of a sensory device (TumorID) and a previously developed tissue ablation device (TumorCNC), was tested using solid, optically-tunable tissue phantoms [20]. We demonstrate that the device can 1) differentiate healthy tissue from neoplastic tissue, 2) generate a cut path along the boundary of the tumor, and 3) execute the generated cut path along the boundary. This procedure is performed in tissue phantoms with a high degree of precision.

## II. Methods

### A. Optical Tissue Phantom Design

The primary testing medium for all experiments was the solid, optically-tunable tissue phantom. These phantoms are variations of existing phantoms created [23]. These phantoms were created to grossly mimic the optical and molecular properties of tumorous and healthy tissues. All phantoms feature agarose (VWR International, Radnor, PA) concentrations at 1.2% (w/v) to provide a solid surface for the phantoms.

Both the tumorous and healthy portions of the tissue phantoms featured the same absorption and reduced scattering properties. The phantoms were designed with an absorption coefficient of $14\,\,cm^{-1}$ and a reduced scattering coefficient of $200\,\,cm^{-1}$ [15]. Similar to Liu *et al.*, $31~\mu M$ of Hemoglobin was used to generate the desired absorption [15]. To achieve these scattering specifications, without using a titanium oxide spheres, 2.5% final concentration of intralipid emulsion (Baxter Healthcare, Deerfield, IL) was used in the phantom [24]. Potential concerns regarding safety when ablating titanium oxide spheres led to the adoption of intralipid. The fluorophores used to replicate endogenous fluorescence observed in the Warburg effect in human tissue were NADH and reduced FAD (Sigma Aldrich, St. Louis, MO). In line with previous studies, the NADH concentration was fixed at 1.2 mM for both tissues; the FAD concentration was set to $1.6~\mu M$ for tumor tissue and $16~\mu M$ for healthy tissue [15].

Previous TumorID studies reveal the ability to resolve differences in FAD concentration in $1.2~\mu M$ increments, between $1.6~\mu M$ and $16~\mu M$ [25]. Additionally, the laser parameters used in previous studies (radiant exposure of $3.5\,\,{J}{cm^{-2}}$) are the same parameters used in this study, and only introduce a 20% reduction in the fluorescence emission maximum after approximately 25 discrete irradiation cycles [25]. The third type of tissue created for the experiment was a fiducial tissue. The fiducial tissue was used to register the location of the phantom tissue for ground truth measurements for the performance of the system. The fiducial was a mixture of 1.2% w/v agarose and 3 mM Perylene-3,4,9,10-tetra-carboxylic dianhydride (PTCDA). PTCDA (Sigma Aldrich, St. Louis, MO) was chosen because it is an organic dye that does not diffuse well into the surrounding water agarose water matrix.

**FIGURE 1.**
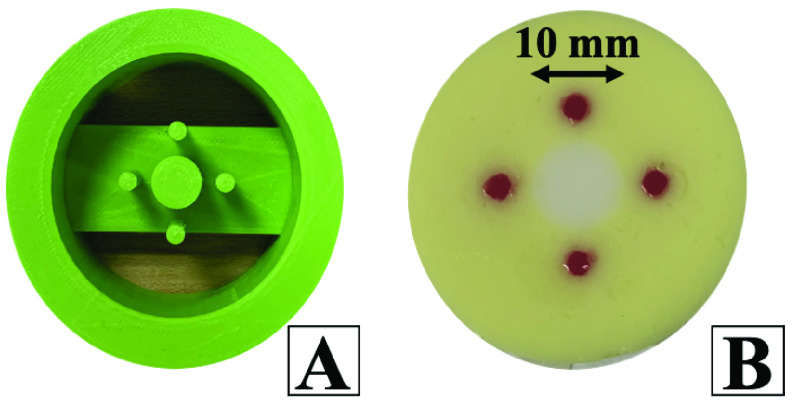
A) 3D printed mold for tissue phantom casting B) Image of completed tissue phantom.

A 3D-printed mold was used to shape and form phantoms accurately and in a repeatable fashion (Fig. 1A) The four posts in the design were used to create four holes where fiducial phantom will eventually be poured. The central post was used to create the hole where the tumor mimicking portion of the tissue phantom was poured. That diameter of the central post was varied in molds at 7.5, 10.0, and 12.5 mm to simulate various tumor sizes. This variation ensured that the size of the tumor mimicking phantom did not affect the performance of the system. The phantom is created by placing the mold on the bench-top, with the posts touching the bench. Healthy tissue phantom was heated and poured into the mold and set aside to cool to room temperature. The 3D mold was gently removed and fiducial tissue phantom was used to fill the four surrounding holes. Finally, the tumorous phantom was used to fill the final hole. The final tissue phantom used in the study is pictured in Figure 1B.

### B. Device Description and Testbed Design

**FIGURE 2.**
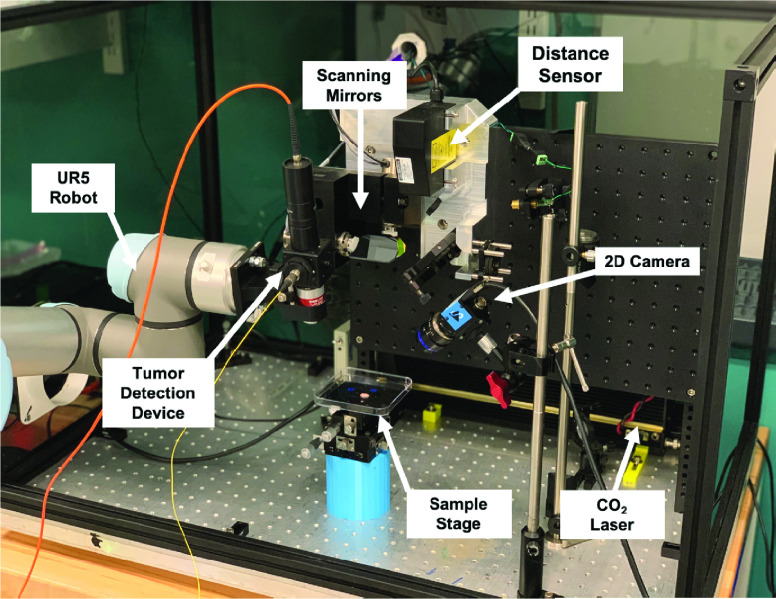
Experimental setup, showing the TumorID positioned with the field of view of the TumorCNC by the UR5 robot.

The TumorID provides the sensory data stream for differentiation of the “healthy” and “tumor” tissue regions within the phantom. Figure 2 depicts the entire experimental testing setup used in the study. A UR5 robotic arm (Universal Robots A/S, Denmark) was utilized to the direct the TumorID detection device in two dimensions over the tissue phantoms. The UR5 enables precise positioning of the TumorID. The distance between the tissue phantom and the last optical element of the TumorID was fixed at 1.7 cm, the working distance of the objective lens. The objective lens is a 0.2 NA, 4X microscope objective (Thorlabs, Newton, NJ). The robot arm was programmed to scan a flat surface with a uniform step size of 1 mm. The 1 mm step size was heuristically chosen to balance spatial resolution with the duration of data collection. In total, a grid of $2.7 \text{ cm} \times 2.7 \text{ cm}$ was collected, for a total of 729 points measurements. The robot was positioned at each point for 2 seconds and the time to move between points was 1 second. This setting ensured that data collected at each point did not affect the following data acquisition phase. Motion planning was performed using inverse kinematics (IK) solvers in the Klampt robotics simulation platform [26].

The TumorCNC is an automated surgical laser system developed by our group for precise soft tissue resection [20]. Elements of the TumorCNC include the cutting laser, distance sensor, and scanning mirrors (Fig. 2). The cutting laser utilized for these studies was a $10.6~\mu m$, 10 W $CO_{2}$ laser (Synrad Inc., Mukilteo, WA). The distance sensor utilized was a triangulation-based distance sensor (Mechanical Technology Inc., Albany, NY). The 2D scanning mirrors galvanometer system (Cambridge Technology, Inc, Bedford, MA) was used to direct lasers from both the distance sensor and $CO_{2}$ laser.

The sample stage was positioned such that the tissue phantom can be observed by the monocular camera and was within the field of view of the TumorCNC and the workspace of the TumorID. The monocular camera (DFK33UP1300 camera sensor and TCL 0814 5 MP 8mm lens) was manually focused with the exposure time adjusted to track the laser spot from the TumorID and the triangulation-based distance sensor.

### C. Description of Experimental Procedure

**FIGURE 3.**
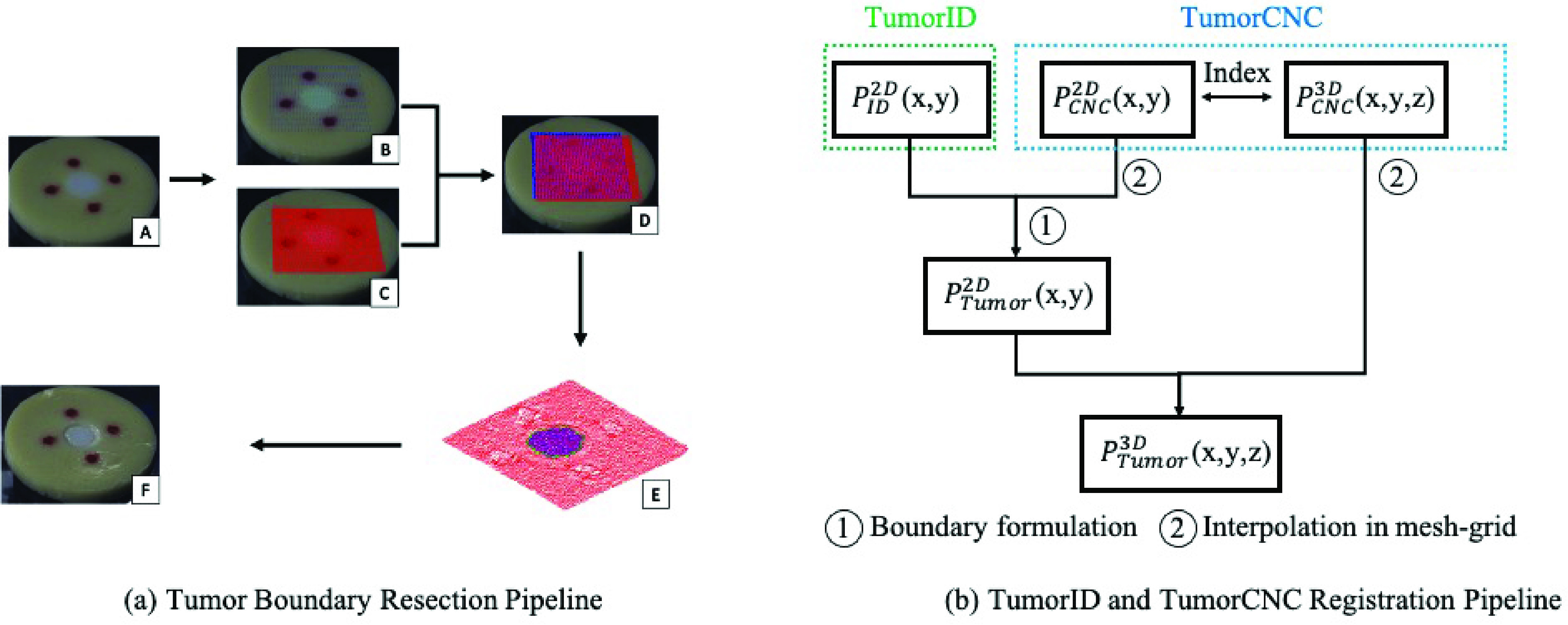
(a) Tumor Boundary Resection Pipeline A) Tissue phantom used for testing B) Superimposed points from TumorID C) Superimposed triangulation-based distance scanner points (red points) D) Overlapping data correlating TumorID data to 3D dimensions E) Tumor tissue classified (in purple) and tumor boundary cut path generated (green) F) Image of the tissue phantom after ablation (b) TumorID and TumorCNC Registration Pipeline - showing how the TumorID and TumorCNC fuse sensory streams to generate a predicted tumor boundary that can be cut by the TumorCNC.

The TumorID is capable of providing valuable sensory data concerning tumor boundary diagnosis. The goal of this research was to find a way to shuttle the tumor boundary diagnostic information *from* the TumorID *to* the TumorCNC, so that there could be automated ablation of the tumor boundary. Figure 3a depicts a representation of the entire surface interrogation workflow for detection and ablation of the phantom tumor boundary. As the fluorescence and distance measurements are in different coordinate systems, we developed a pipeline to transform the data points in a unified frame defined in the TumorCNC system. With both system frames registered to one another using the common frame of the monocular camera, an imaging path for the TumorID and a cutting path could be calculated in the unified frame. Specifically, the steps of the pipeline are:

**FIGURE 4.**
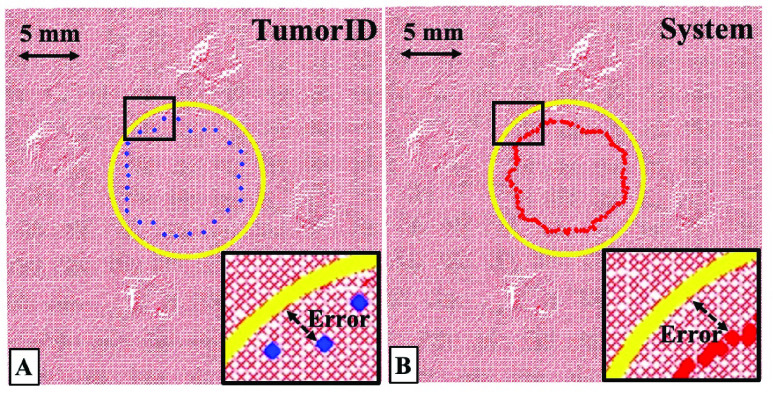
A) A notional diagram for how error is measured for the performance of the TumorID. Distance between estimated tumor boundary (blue) of the TumorID and the ground truth tumor boundary (yellow) is the foundation of the error measurements given. B) Similar to the TumorID measurement, the System measurement is a measure for performance of the total system (TumorID and TumorCNC). Distance between the measured center of the ablated region (red) and the ground truth boundary (yellow) is the foundation of the error measurements calculated for the total system.

#### 1) Raster Scanning and Data Collection

The TumorID system firstly performed a raster scan and collected the fluorescence data at the tissue surface. The monocular camera captured the color image for the visible 405 nm laser spots with a low exposure time setting (reduce the halo effects of over exposure). We then obtained the pixel location, in 2D coordinates, of the laser centroids using image segmentation methods in the CIELAB color space [27], which is referred as $P_{ID}^{2D}(x,y)$. For each fluorescence measurement, the maximum intensity value was chosen and used as the sole parameter for classification. Therefore, we were able to label the pixel coordinate in the image classified as either tumorous or non-tumours data based on a pre-defined intensity threshold.

Secondly, the TumorCNC system scans the tissue surface based on its internal laser scanning setup that contains a 3D distance sensor and a two-axis galvanometric mirror system [31]. The distance measurement was used to calculate the 3D coordinate of the point where the laser was incident to the surface; the dynamic model utilized for this task has been described previously [31]. The 3D coordinate was also recorded and denoted as $P_{CNC}^{3D}(x,y,z)$. For the scanning portion, a step size of 0.25 mm was used between each laser spot. Similarly, the camera captured the laser spot emitted by the distance sensor and the 2D pixel coordinate is denoted as $P_{CNC}^{2D}(x,y)$.

#### 2) TumorID and TumorCNC Registration

The fluorescence and distance measurements were measured in different coordinate systems and thus, we defined a unified global frame as the same one in the TumorCNC system [28], [31]. This global frame was defined at the center of the scanning mirror and the positive direction of the Z axis was perpendicular to the ground. As it is difficult to compare actual contours in a 3D coordinate system, and $P_{ID}^{2D}(x,y)$ and $P_{CNC}^{2D}(x,y)$ are measured at the same phantom surface, we chose to transform all the points to a 2D projected plane. In this system, this transformation was to remove the value in the Z axis in the distance measurements, i.e. $(x,y, {z})\rightarrow (x, y)$. With this definition, The TumorID data can be registered to the global frame for tumor boundary formulation and laser cutting.

#### 3) Tumor Boundary Generation

The tumor boundary is formulated by the fluorescence measurement and registered to the TumorCNC for laser cutting. Specifically, each fluorescence measurement was classified as two classes (tumorous or healthy) and labeled as different color in the corresponding pixel coordinate in Fig. 3a. These fluorescence measurements were used to formulate an edge by using the “Boundary” function in MATLAB, which is an implementation of a method using alpha shapes [29]. The 2D coordinates of the distance measurements located inside this fluorescence-boundary can be found using the built-in function “Inpolygon” in MATLAB. These points can be used formulate an initial tumor boundary. As the original fluorescence-boundary has fewer sample data points around the edge, because of the large scanning step size (i.e. 1.0 mm for TumorID), the new boundary formulated by the distance measurements has a greater number of points to define the boundary.

#### 4) Laser Cutting Path From Tumor Boundary

To generate a smooth and less jerky cutting path for boundary tissue removal, the initial boundary was refined in a $150\times150$ interpolated mesh-grid with more data points around the edge, which reduces the risk of cutting incorrect tissue region in the phantom. Notably, the new tumor boundary was mapped to the 2D global frame and denoted by $P^{2D}_{Tumor}(x,y)$. As the 2D and 3D distance measurements share the same index, we can find the correspondences of $P^{2D}_{Tumor}(x,y)\vphantom {\sum _{R_{R_{r}}}}$ and the new 3D coordinates are referred as $P^{3D}_{Tumor}(x,y,z)$, which can be used to formulate a 3D laser cutting trajectory. A simplified pipeline of generating the 3D laser cutting path, by registering the two systems with one another, is illustrated in the Fig. 3b.

#### 5) Post-Ablation Laser Profile

**FIGURE 5.**
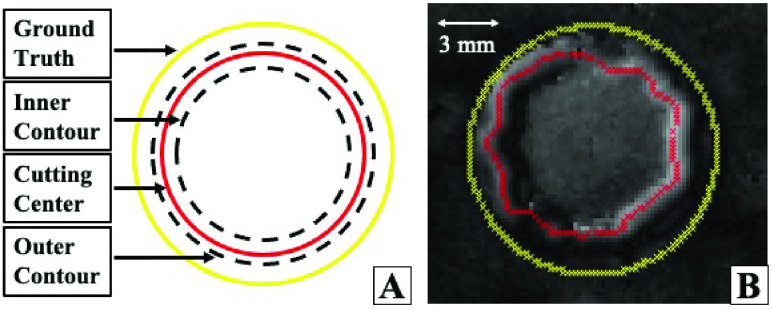
A) Diagram of the ablated cut and how the cutting contour is calculated for system measurement (yellow ground truth contour, red center cutting contour, black dashed line representing inner and outer cutting contours) B) Difference of data from triangulation-based distance sensor, with each pixel denoting the depth of change. The yellow line represents the ground truth of the tumor boundary and the red line represents the cutting contour, which is the center of the ablated “well.”

Given the laser 3D trajectory, the TumorCNC via the $CO_{2}$ laser beam targeted each point in the cutting path by precisely controlling the scanning mirrors. The laser parameter was adapted to this experiment by setting the PWM (pulse width modulation) signal as 40% (power increases from 0% to 100%), spot size as 0.80 mm and mirror frequency as 20 points per second. Before and after the laser ablation, the TumorCNC used the distance sensor to conduct a raster scan on the phantom surface to generate a 3D point cloud. The pre-scan and post-scan point cloud are defined in the global coordinate system and projected to a 2D plane. The Z coordinate value is denoted as a pixel intensity which shows the depth of change. Therefore, the difference of the pre-scan and post-scan point cloud were calculated by subtracting the pixel intensity in the corresponding coordinate. Figure 5B shows the difference of depth between pre-scan and post-scan point cloud.

### D. Experimental Error Measurement

Error measurements enabled us to evaluate how well the integrated system preformed at autonomously identifying and targeting the boundary of the tumor. We utilize root mean square error (RMSE) measurements and maximum error measurements (ME) to compare the expected contour of points versus the actual contour of points. In this study, we use two different error measurement metrics to evaluate how well the TumorID can detect the tumor boundary and TumorCNC can remove the tissue identified by the fluorescence-guided boundary.

#### 1) Ground Truth

As shown in Fig. 1B, the fiducial tissue was designed to provide ground truth measurements. The fiducial tissue had a different spectroscopic signature than the other parts of the phantom. The fiducial pixel coordinate was found in the color image. Similarly, these fiducial pixel coordinates were transformed to the global coordinate frame by looking for the nearest point in the distance measurement. Using the information from the 3D CAD file, the ground truth in this study was defined as the center of the tumorous tissue and the size of the radius. The center and radius values were used to formulate a ground truth boundary contour by the new fiducial coordinates, referred to as $Q_{GT}$.

#### 2) TumorID Error Measurement

In the cutting path pipeline, the process of transforming the tumorous edge coordinates of the fluorescence measurements to the global coordinate system, referred to as $P_{ID-EDGE}$, is outlined. This original fluorescence-boundary has fewer data points at the edge because of the larger scanning step size for TumorID. $P_{ID-EDGE}$ was used to evaluate the performance of the fluorescence-guided detection procedure by calculating the difference to the ground truth $Q_{GT}$.

#### 3) System Error Measurement

During the removal of the tumor boundary, the ablation generated material removal in the form of an ablation crater. This crater was denoted as the change of depth for the distance measurement. We used the ablation center to represent the actual ablation contour. This actual contour is compared to the ground truth of the phantom boundary $Q_{GT}$ to determine how well the system removed the tumor boundary.

The actual contour was estimated based on the distance measurements between the pre-scan and post-scan point cloud. As discussed in the previous section, the post-ablation laser profile was transformed to a 2D projected plane and each pixel intensity value shows the depth of change in the Z axis. This creates a difference map as shown in Fig. 5B. The pixel regions with intensity greater than a defined threshold (the threshold is related to the change of depth) were classified as the coordinate inside the laser crater. The inner and outer contours were localized by this pixel region, and the cutting center was defined as the center between the inner and outer contours. These post ablation centers formed a 3D trajectory and for each point in $Q_{GT}$ we found the closest point in this post-ablation contour, which was referred to as $P_{POST}$. The RMSE and ME were estimated based on the difference between the $P_{POST}$ and $Q_{GT}$.

#### 4) General Error Measurements

For the error comparison, two point sets were used. One point set represents the actual recorded data and another one is the expected data associated with the ground truth values. Assume the two point sets are denoted by $P =\{ p_{1}, p_{2}, \cdots, p_{n} \}$ and $Q = \{ q_{1}, q_{2}, \cdots, q_{n} \}$ with the same number of points. Based on this definition and we calculated the RMSE and ME by:

\begin{align*}&\hspace {-1pc} RMSE \\&{ = \sqrt {\frac {1}{n} (||p_{1} - q_{1}||_{2}^{2} + ||p_{2} - q_{2}||_{2}^{2} + \cdots + ||p_{n} - q_{n}||_{2}^{2})} }\end{align*}

Max Error describes the greatest error observed:

\begin{align*}&\hspace {-1pc}Max Error \\&= max \{ ||p_{1} - q_{1}||_{2}, ||p_{2} - q_{2}||_{2}, \cdots, ||p_{n} - q_{n}||_{2} \}\end{align*}

In this study, the TumorID and the system error measurement were estimated based on the difference between $P_{POST}$ and $Q_{GT}$, and $P_{ID-EDGE}$ and $Q_{GT}$, respectively.

## III. Results

### A. Total System Performance

**FIGURE 6.**
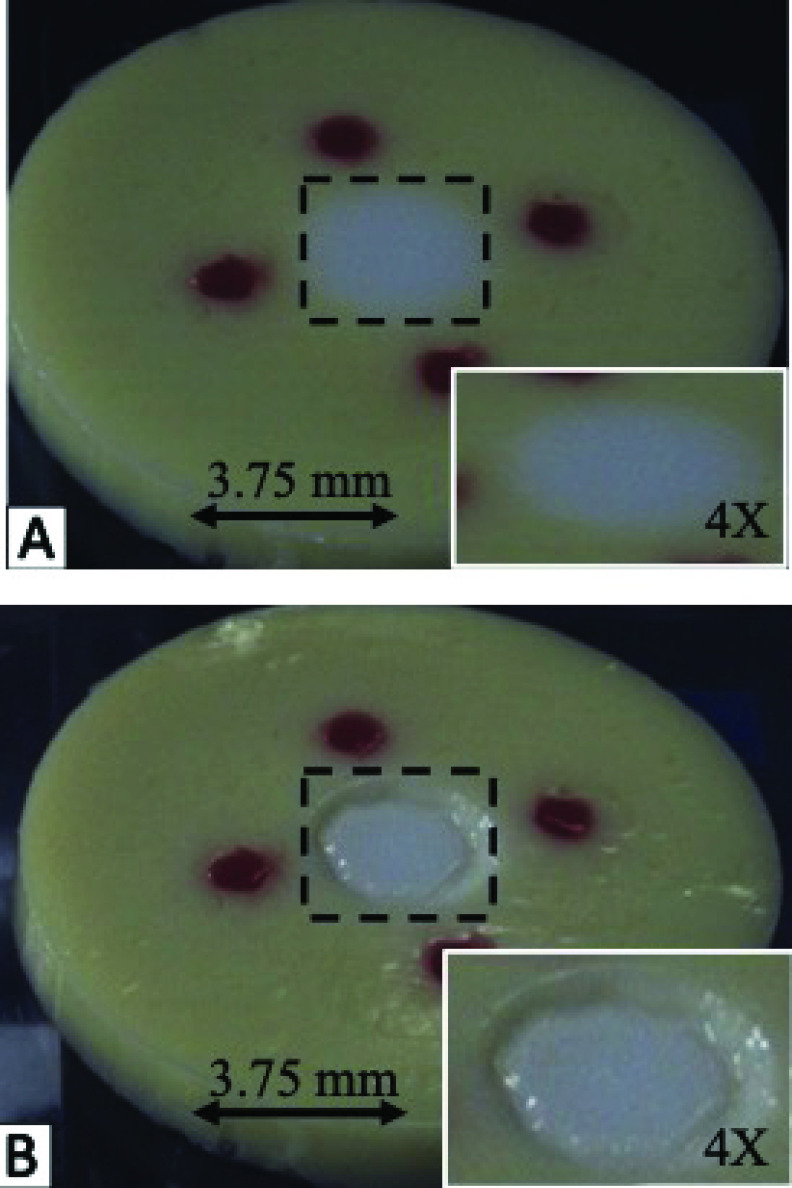
Tissue phantom before (A) and after (B) ablation targeting the tumor boundary. Tumor regions can be seen in greater detail in respective insets. Only the estimated boundary was ablated, the “bulk” of the tumor was not ablated.

**FIGURE 7.**
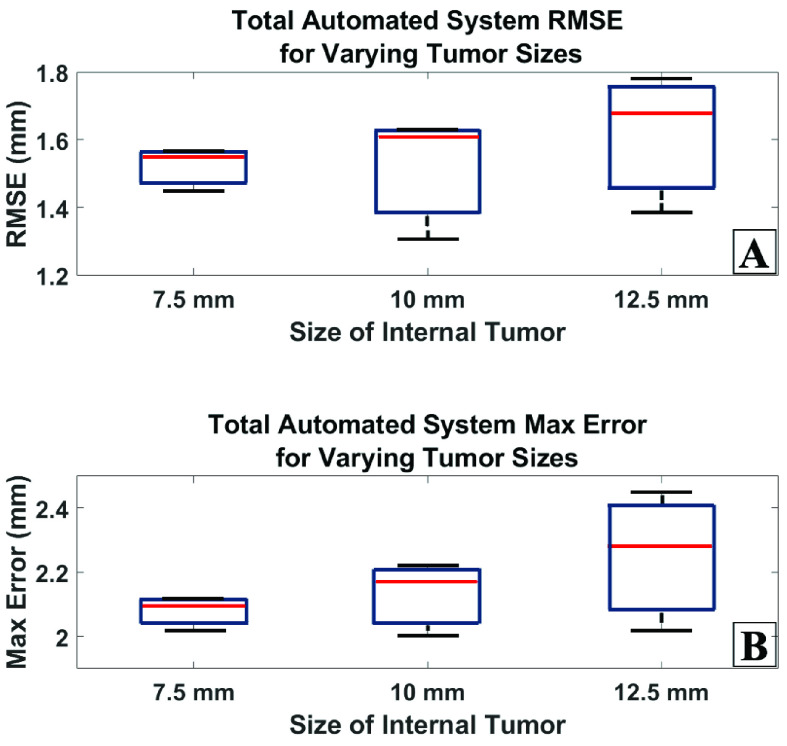
A) System RMSE for varying tissue phantom tumor sizes B) System max error for varying tissue phantom tumor sizes.

Figure 6A shows a tissue phantom before boundary ablation and Figure 6B shows the tissue phantom after boundary ablation. The RMSE and max error are plotted in Figure 7A and Figure 7B, respectively. The system performance was plotted for each tumor diameter size. Three phantoms were measured for each tumor diameter size. The total system average RMSE for each of the 7.5, 10.0, and 12.5 mm phantoms is 1.52, 1.52, and 1.62 mm, respectively. For all phantoms tested, the total system RMSE fell between 1.31 mm and 1.78 mm. The total system average max error for each of the 7.5, 10.0, and 12.5 mm phantoms is 2.08, 2.13, and 2.25 mm, respectively. For all phantoms tested, the total system max error fell between 2.00 mm and 2.45 mm.

### B. TumorID Performance

**FIGURE 8.**
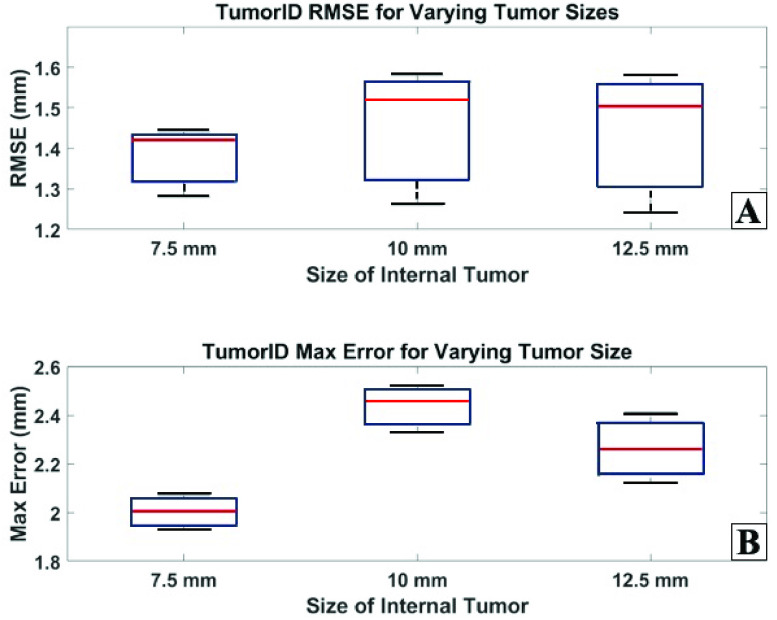
A) TumorID RMSE for varying tissue phantom tumor sizes B) TumorID max error for varying tissue phantom tumor sizes.

Figure 8 shows the RMSE and max error for the TumorID portion of the experiment. The TumorID element measures the error between the ground truth tumor boundary and the projected tumor boundary based on the TumorID. The TumorID average RMSE for each of the 7.5, 10.0, and 12.5 mm phantoms is 1.38, 1.45, and 1.44 mm, respectively. For all phantoms tested, the TumorID RMSE fell between 1.24 mm and 1.58 mm. The TumorID average max error for each of the 7.5, 10.0, and 12.5 mm phantoms is 2.01, 2.44, and 2.27 mm, respectively. For all phantoms tested, the TumorID max error fell between 1.93 mm and 2.52 mm.

## IV. Discussion

The ability to rapidly discriminate between pathological and normal tissue during the course of a brain tumor resection is of utmost importance for maximal resection of the tumor, while minimizing damage to surrounding delicate neural structures. Coupling real-time intraoperative imaging modalities such as fluorescence-guided surgery, with precise robotically controlled soft tissue removal, opens an avenue for ultra-precise, automated soft tissue surgery. In this study, we presented a method by which an automated device can target tumor mimicking phantom tissue and then remove this pathologic tissue utilizing laser-guided surgery.

We found that the combined efforts of the TumorID endogenous fluorescence imaging system and the TumorCNC automated laser soft tissue resection platform were capable of this precise, automated ablation. Here, we demonstrated the maximum average total system RMSE for such resection was 1.62 mm. Despite the performance of the system, the reported maximal precision for a neurosurgeon under contrived *ex vivo* optimal microscopic conditions is maximally 0.289 mm [30]. Therefore, while this system does approximately remove the tumor boundary, there is still significant room for improvement in order to promote the device to a level comparable to the maximal reported precision boundary recorded by human surgeons.

Performance does not vary significantly between tumor sizes between 7.5 and 12.5 mm, a range that has clinical relevance with regards to frequent brain tumor sizes. All average RMSE values fall within a 0.10 mm range of each other. The greatest average ME for the system performance is equal to 2.25 mm. This indicates that on average, the greatest deviation between the ground truth tumor boundary and the ablated region was 2.25 mm. The standard deviation for the RMSE and ME for each phantom is no greater than 0.20 mm. This indicates that the device is capable of removing tissue repetitively, with little variation. Further work is needed to ensure that significant deviations from the target do not occur, as that would have significant clinical implications in neurosurgery.

The TumorID findings in isolation, even without TumorCNC ablation capability, offer insights into how much of the system error is related to the diagnostic arm of the device. The TumorID contributed to a large share of the error in the total system. The greatest average RMSE and average ME for the TumorID was 1.45 mm and 2.44 mm, respectively. There are potentially two areas for improvement that could drastically increase the performance of the integrated system. First, the TumorID needs a more intelligent searching strategy. The current data capturing strategy relies on a brute force approach that collects data in a grid of uniformly spaced points. We hypothesize that a more intelligent search method, one that prioritizes a greater density of points around the tumor boundary, will yield a more accurate classification of tumor versus healthy tissue. We hypothesize a greater density of points will generate greater effective spatial resolution.

In addition to increasing the number of points at the boundary, we hypothesize that decreasing the number of points in the center will decrease the amount of time taken for data collection. Each point requires two seconds for data acquisition and one second between points. By decreasing the number of points, seconds will be shaved while preserving the fidelity of the data collected by the TumorID. This will ensure that there is a temporal advantage to using our device (in addition to a precision and accuracy advantage).

The second strategy that we believe will improve the TumorID classification is the introduction of a more intelligent classifier. The current classifier is a basic, binary classifier that uses the single parameter of maximum fluorescence intensity to divide tissue into predicted groups of tumorous or healthy tissue. We believe a multi-class classifier will lead to more accurate diagnostic prediction and reduce the prediction error. The binary classifier fidelity begins to degrade around the boundary, where a greater distribution of intensities exist.

The device and testing platform are useful tools that aid in demonstrating that automated, fluorescence-guided tumor resection is possible using a mix of photonic solutions. However, there were a number of sources of error associated with the construction of the device and the fidelity of the measurements associated with the experimental testbed.

Error associated with the design of the device can be attributed to the fact that the TumorID and TumorCNC do not share an optical axis. An optical axis will likely reduce system error due to the lack of system registration. This device requires a direct line of sight to tumorous tissue in the brain. In addition to registration related error, the type of laser used in the experiment introduces error. The size of the $CO_{2}$ laser spot (0.80 mm) introduces error associated with how precise the cut can be with respect to the tumor boundary. The size of the 405 nm laser spot (0.75 mm) is not as consequential to error, due to the ability to take overlapping measurements.

Error associated with the testbed and experimental procedure include the quantification of the laser cut. Potential error is introduced into the quantification of system performance due to the interaction between the $CO_{2}$ laser and the tissue phantom. Despite minimal drift in power, and a planning algorithm that reduces errant irradiation, there is still a chance that a more precise laser could marginally increase performance. This is specifically due to the tissue phantom testing medium and that small differences in laser power or beam irradiation have the potential to change the perceived performance of the system. Finally, the use of fiducial phantoms to create a ground truth does introduce some transient error into the system. We rely on a spectroscopic system (TumorID) to detect a different spectral signature and then use tumor diameter information from the CAD file to estimate the boundary. Although the errors that result from this method will only be tens of microns, it will present a challenging scenario where progress will potentially be constrained due the intrinsic error of the tissue phantom.

Additionally, when considering the performance of the system, it is important to note that generally the device would “undershoot” the true margin. That is to say, there was tumor-mimicking phantom tissue between the ablated boundary and the ground truth boundary. While this is preferable in neurosurgery, where healthy brain tissue preservation is maximized, it does present clear room for improvement for the device if used in other surgeries where negative margins are acceptable.

In addition to performance, it is also important to consider how this device would fit into current neurosurgical workflows. We see this device as a ubiquitous tool that could be used in a variety of ways to assist the surgeon. For instance, the device could be used at the start of the surgery to help constrain the working area for the surgeon and simultaneously increase the extent of resection. Additional improvements on the device are needed to realize successful incorporation of the unified TumorID & TumorCNC into a typical neurosurgical workflow. Specifically, time is currently a considerable constraint.

Total time taken by the device, from sensing to removal, for a single solid tissue phantom tumor boundary, takes approximately an hour (approximately 65 minutes). Major contributors to total time taken include the process of scanning the region of interest by the TumorID (approximately 35 minutes) and the high fidelity scan by the triangulation-based distance sensor (approximately 30 minutes). The formulation and execution of a laser cut path only takes 3 seconds, so that time is negligible. According to stakeholder analyses conducted by our lab, we believe that a sixfold reduction in time will yield a performance that would be suitable for incorporation into a typical neurosurgical workflow that could benefit from this device. Although not currently a crucial need, eventually a microscope objective with a longer working distance will be needed to increase the distance between the TumorID and the tissue in the sterile field. We are confident that custom fabrication of a microscope objective could increase the working distance to 3 cm, with comparable NA and magnification for the required wavelengths.

In the future, the device will also need to be adapted to allow access to deep seated tumors or those with complex three dimensional geometries within the parenchyma of the brain. The current approach utilizes an underlying assumption that the tumors are accessible on the brain surface or direct line to an exposed resection cavity. We anticipate that future work will include modifications of the underlying hardware to facilitate dynamic positioning relative to the resection cavity or even non-linear approaches to deep seated lesions.

## V. Conclusion

Application of image-guided robotics for neurosurgical tumor removal stands to increase the accuracy and precision of neurosurgical procedures, reduce cognitive burden for the surgeon and care team, and improve surgical outcomes for patients through more efficient and precise surgery. We have created a fluorescence-guided tumor resection platform that relies on fluorescence feedback to autonomously remove targeted tumor tissue. This completely non-contact system is capable of removing the tumor boundary of a tissue phantom with an average RMSE of approximately 1.55 mm and an average max error of approximately 2.15 mm. There is no difference in performance based on the size of the tumor mimicking tissue phantom boundary. Future directions include creating a more intelligent TumorID search strategy to increase the density of points around the boundary, and the development of a more sophisticated classifier to predict tissue type around the tumor boundary.
